# Supplementary material for: Genome-Wide Identification of Wheat Gene Resources Conferring Resistance to Stripe Rust
Source: Plants (Basel). 2025 Jun 19;14(12):1883. doi: 10.3390/plants14121883 (PMC12196567; doi:10.3390/plants14121883)
Supplement: Supplementary file 1 [file plants-14-01883-s001.zip › Supplementary Figures-to Plants.pdf]

| | Genes | 1 | 2 | 3 | 4 | 5 | 6 | 7 | 8 | 9 | 10 | 11 | 12 | 13 | 14 | 15 | 16 | 17 |
|---|---|---|---|---|---|---|---|---|---|---|---|---|---|---|---|---|---|---|
| 1 | TraesCS4A01G272000.1 | 100 | | | | | | | | | | | | | | | | |
| 2 | AtPAP8 | 28.05 | 100 | | | | | | | | | | | | | | | |
| 3 | BdPAP8 | 25.71 | 56.34 | 100 | | | | | | | | | | | | | | |
| 4 | TraesCS2B02G452300.1 | 25.96 | 56.87 | 89.5 | 100 | | | | | | | | | | | | | |
| 5 | TRITD2Bv1G212300.1 | 25.96 | 56.87 | 89.5 | 100 | 100 | | | | | | | | | | | | |
| 6 | TRIDC2BG065980.1 | 25.59 | 56.87 | 89.5 | 100 | 100 | 100 | | | | | | | | | | | |
| 7 | HvPAP8 | 25.96 | 57.35 | 89.5 | 94.52 | 94.52 | 94.52 | 100 | | | | | | | | | | |
| 8 | TraesCS2D02G428800.1 | 26.44 | 57.82 | 89.95 | 95.89 | 95.89 | 95.89 | 97.26 | 100 | | | | | | | | | |
| 9 | AET2Gv20951700.2 | 26.44 | 57.82 | 89.95 | 95.89 | 95.89 | 95.89 | 97.26 | 100 | 100 | | | | | | | | |
| 10 | TRIDC2AG062110.1 | 26.92 | 55.92 | 89.5 | 96.35 | 96.35 | 96.35 | 94.98 | 96.8 | 96.8 | 100 | | | | | | | |
| 11 | TuPAP8 | 26.92 | 56.4 | 89.95 | 96.8 | 96.8 | 96.8 | 95.43 | 97.26 | 97.26 | 98.63 | 100 | | | | | | |
| 12 | TraesCS2A02G431000.1 | 26.92 | 56.87 | 90.41 | 97.26 | 97.26 | 97.26 | 95.89 | 97.72 | 97.72 | 99.09 | 99.54 | 100 | | | | | |
| 13 | TRITD2Av1G250370.1 | 26.92 | 56.87 | 90.41 | 97.26 | 97.26 | 97.26 | 95.89 | 97.72 | 97.72 | 99.09 | 99.54 | 100 | 100 | | | | |
| 14 | ObPAP8 | 25 | 54.25 | 84.93 | 82.03 | 82.03 | 82.03 | 83.41 | 82.95 | 82.95 | 83.41 | 83.87 | 83.41 | 83.41 | 100 | | | |
| 15 | ZmPAP8 | 24.4 | 55.45 | 84.65 | 83.1 | 83.1 | 80.18 | 82.63 | 82.63 | 82.63 | 82.16 | 83.1 | 82.63 | 82.63 | 85.58 | 100 | | |
| 16 | SiPAP8 | 25.12 | 56.25 | 85.12 | 82.16 | 82.16 | 79.28 | 84.04 | 82.63 | 82.63 | 83.1 | 83.1 | 82.63 | 82.63 | 86.98 | 85.71 | 100 | |
| 17 | SbPAP8 | 24.29 | 57.08 | 87.16 | 83.8 | 83.8 | 82.27 | 84.26 | 84.26 | 84.26 | 83.8 | 83.8 | 84.26 | 84.26 | 85.32 | 92.24 | 93.12 | 100 |

**Figure S1.** Sequence similarity of *PAP8* orthologs and *TaFBN4* across plant species.

Protein sequences are aligned to analyze homology among PAP8 orthologs and TaFBN4 from *Triticum aestivum* (common wheat) and related species. Accession IDs are provided for each sequence, including: BdPAP8 (XP_003580504.1, *Brachypodium distachyon*), HvPAP8 (XP_044967710.1, *Hordeum vulgare*), ObPAP8 (XP_006652787.1, *Oryza brachyantha*), SiPAP8 (XP_004976737.2, *Setaria italica*), SbPAP8 (XP_002448489.1, *Sorghum bicolor*), ZmPAP8 (NP_001338050.1, *Zea mays*), AtPAP8 (NP_001154722.1, *Arabidopsis thaliana*), TuPAP8 (XP_048558974.1, *Triticum urartu*), AET2Gv20951700.2 (*Aegilop tauschii*), TRITD2Bv1G212300.1 (*T. durum*), TRITD2Av1G250370.1 (*T. durum*), TRIDC2BG065980.1 (*T. dicoccoides*), TRIDC2AG062110.1 (*T. dicoccoides*), TraesCS2A02G431000.1 (*T. aestivum*), TraesCS2D02G428800.1 (*T. aestivum*), TraesCS2B02G452300.1 (*T. aestivum*). Sequence data were sourced from NCBI (https://www.ncbi.nlm.nih.gov) or WheatOmics 1.0 (http://202.194.139.32/).

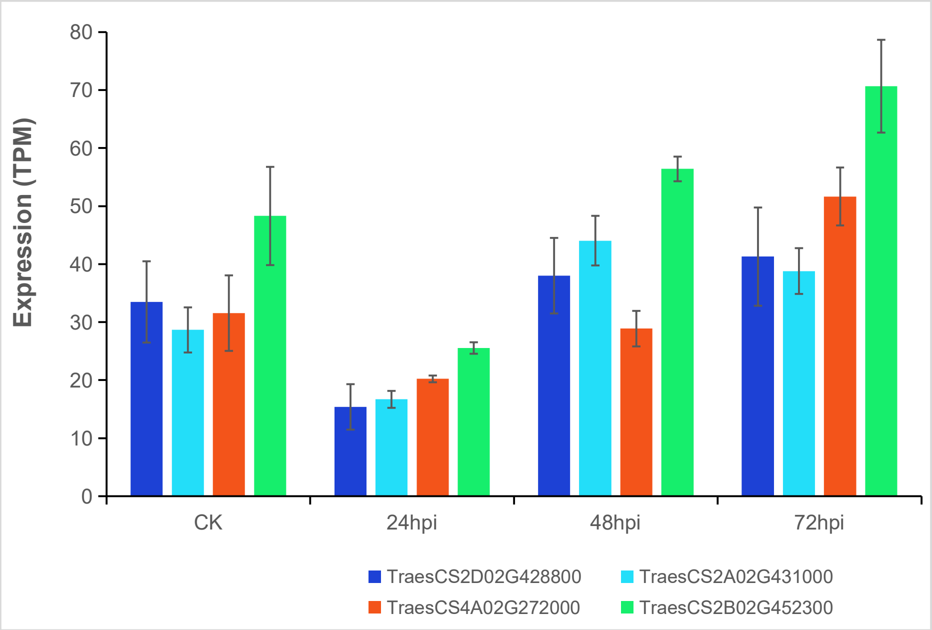


**Figure S2.** Expression patterns of *TaFBN4* and *TaPAP8* genes in response to the Puccinia striiformis f. sp. tritici race CYR31 infection.

Relative expression levels of *TaFBN4* (*TraesCS4A02G272000*), *TaPAP8* homologs (*TraesCS2A02G431000*, *TraesCS2B02G452300*, and *TraesCS2D02G428800*) are analyzed at 24, 48, and 72 hours post-inoculation (hpi) with CYR31, compared to non-inoculated control (CK). Expression data were derived from WheatOmics (http://202.194.139.32/).

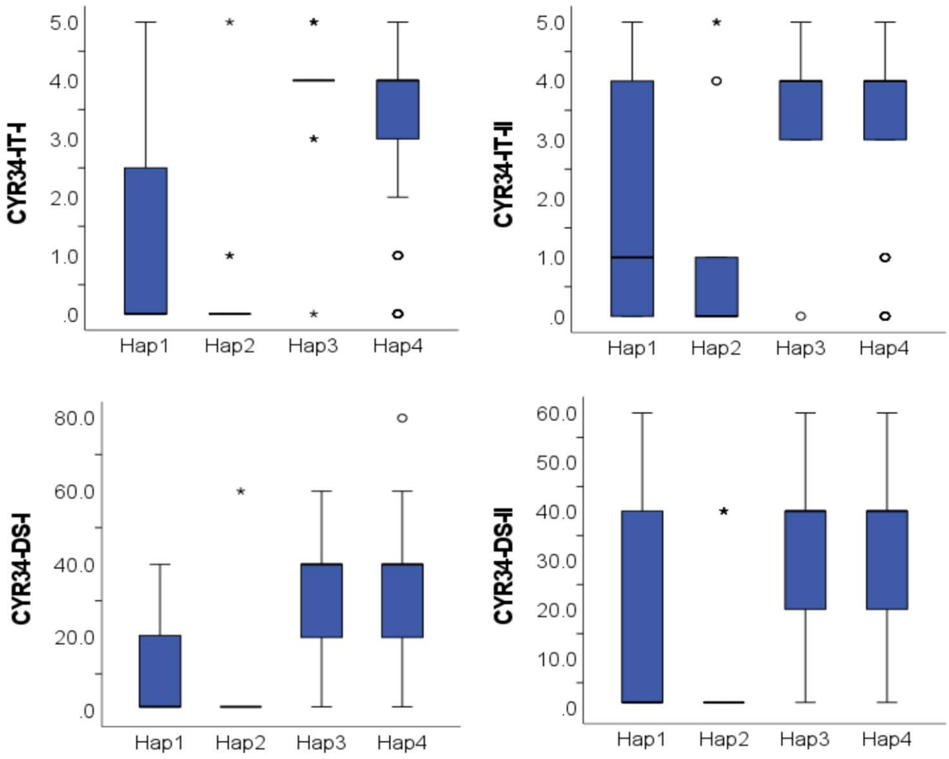


**Figure S3.** Phenotypic effects of *QYr.baafs-1D.2* haplotypes onon disease severity (DS) and infection type (IT) against the CYR34 race.

Haplotypes (Hap1 – Hap4) were evaluated for resistance to CYR34 at the seedling stage. DS values (bottom panel) represent the percentage of infected tissue, and IT values (top panel) indicate infection severity. Data are shown as means ± standard deviation (SD). Statistical significance among haplotypes was assessed using one-way ANOVA followed by Duncan's test ($P < 0.05$).

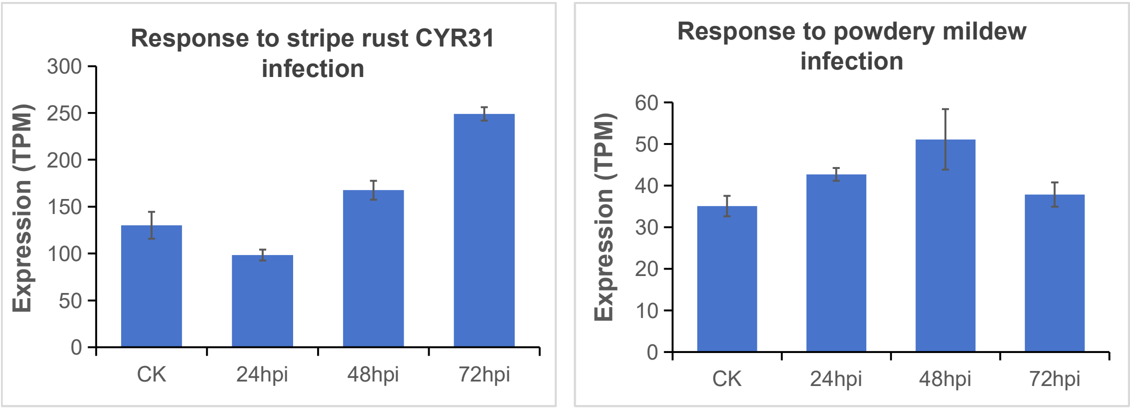


**Figure S4.** Expression of candidate gene *TraesCS1D02G112300.1* in *QYr.baafs-1D.2* region during biotic stress.

Transcript levels of *TraesCS1D02G112300.1* (encoding 6-phosphofructo-2-kinase/fructose-2,6-bisphosphatase) are measured after inoculation with stripe rust or powdery mildew. Expression data from WheatOmics (http://202.194.139.32/) show upregulation at 24, 48, and/or 72 hpi compared to non-inoculated control (CK).

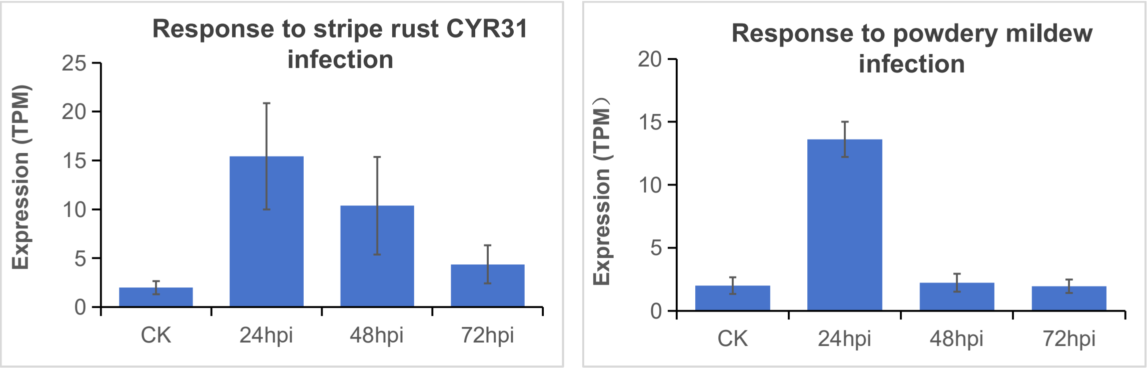


**Figure S5.** Expression of candidate gene *TraesCS1B01G281200.1* in *QYr.baafs-1B.4* region during biotic stress.

Relative expression of *TraesCS1B01G281200.1* (encoding a transmembrane protein) was analyzed following infection with stripe rust (CYR31) or powdery mildew. Data from WheatOmics (http://202.194.139.32/) show induced expression at 24, 48, and 72 hpi compared to non-inoculated control (CK), supporting its role in disease resistance.

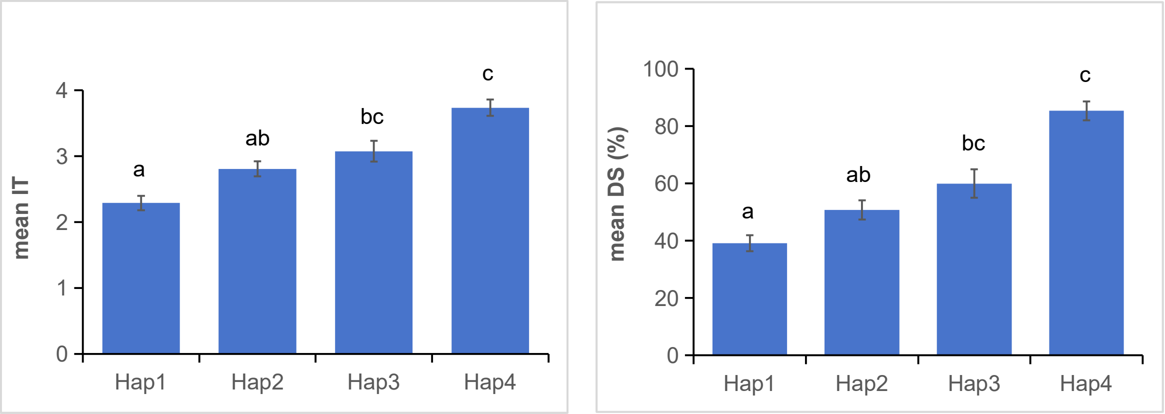


**Figure S6.** Phenotypic effects of *QYr.baafs.1B.4* haplotypes on disease severity (DS) and infection type (IT) against mixed stripe rust races at adult-plant stage.

Haplotypes (Hap1–Hap4) were evaluated across three field environments. DS values (left panel) and IT values (right panel) represent mean responses to mixed *P. striiformis* races. Data are shown as means ± SE. Statistical significance among haplotypes was determined using one-way ANOVA followed by Duncan's test (P < 0.05; different letters indicate significant differences).

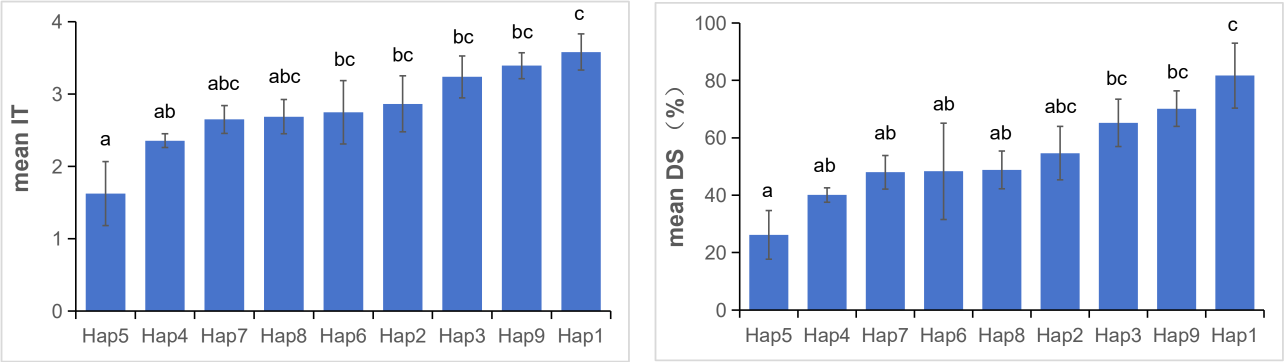


**Figure S7.** Phenotypic effects of *QYr.sicau-6BS* haplotypes on disease severity (DS) and infection type (IT) against mixed stripe rust races at adult-plant stage.

Nine haplotypes derived from 14 resistance-linked SNPs were assessed for resistance to mixed P. striiformis races. DS and IT values (mean ± SE) are shown, with Hap5 exhibiting the highest resistance. Statistical analysis via one-way

ANOVA and Duncan's test ($P < 0.05$) identified significant differences among haplotypes (different letters denote significance).

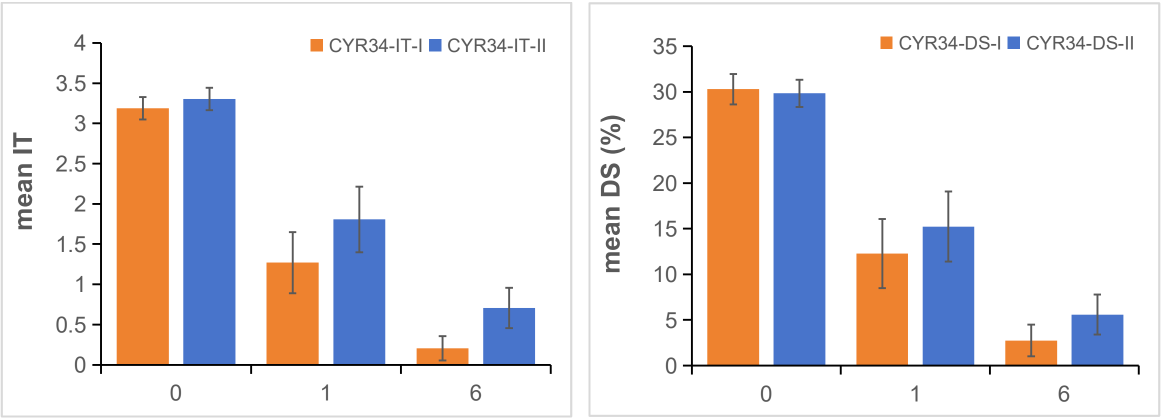


**Figure S8.** Correlation between the number of favorable haplotypes (FHs) and stripe rust resistance to the CYR34 race.

Disease severity (DS, left panel) and infection type (IT, right panel) were plotted against the number of FHs (zero, one, and six) for seven CYR34-associated QTL. A significant inverse correlation ($P < 0.001$) was observed, indicating that increased FHs correlate with reduced disease symptoms, supporting the cumulative effect of favorable alleles in resistance breeding.
